# Talent Management, Affective Organizational Commitment and Service Performance in Local Government

**DOI:** 10.3390/ijerph17134827

**Published:** 2020-07-04

**Authors:** Roberto Luna-Arocas, Francisco J. Lara

**Affiliations:** 1 Faculty of Economics, University of Valencia, Business Management Department, 46022 Valencia, Spain; 2 Management Department, CUA—The Busch School of Business, Department of Management, Washington, DC 20064, USA; laraf@cua.edu

**Keywords:** talent management, local government, organizational commitment, service performance

## Abstract

Talent management (TM) is a fundamental issue for both private and public sector companies. This study analyzes the impact of TM on service performance (SP) and the mediating role of affective organizational commitment (AOC). We analyze a sample of 104 local government employees with three measures of TM, AOC and SP. The mediation hypothesis of AOC was also raised in the study using Baron and Kenny’s methodology and Hayes PROCESS. The results reveal how AOC is a total mediating variable in the causal relationship TM → SP. This study is cross-sectional. Common-method bias is controlled in the study. The results involves a concern for improving services through the professionals who provide them, which in turn entails managing people in a way that is different, more flexible, less bureaucratic, and more client- or citizen-oriented. Given the scant research exploring the role of talent management in public services, this article offers valuable insights for scientific literature and practitioners in the public administration.

## 1. Introduction

The current highly competitive situation in organizations implies having a deep understanding of the new social and economic challenges [1], which not only affect private sector companies but also have a major impact on those in the public sector. Faced with this uncertain scenario, talent management (TM) emerges as a crucial strategy in organizations to meet their needs for flexibility, competitiveness and efficiency [2,3]. Indeed, the key to competitiveness lies in the management of talent, not only in its formulation but in its effective implementation, bridging the gap in the literature between theory and practice [4].

This is why, in recent years, finding talented people has become one of the main concerns of organizations in an effort to meet both the performance and the potential required for the immediate future. For this reason, it has aroused so much interest amongst professionals and academics involved in the area [5,6]. This entails considering how to attract, develop, and secure the loyalty of talented people so that they fulfil the organization’s objectives with excellence [7], or as Ally Weeks put it in the factsheet of the Chartered Institute of Personnel and Development (CIPD) [8]: “talent management is the systematic attraction, identification, development, engagement, retention and deployment of those individuals who are of particular value to an organization, either in view of their ‘high potential’ for the future or because they are fulfilling business/operation-critical roles”.

In this way, talent management not only proves key in the private but also in the public sector [6,9,10,11,12,13,14,15,16,17,18]. In fact, the war for talent is the main agenda for public services worldwide [19,20,21].

Research on talent management is still in its infancy and, as such, there are few studies on this subject in the domain of public administration [5,20]. In fact, most scientific studies have been carried out in private sector organizations and multinationals [22]. In addition, practical interest in talent management has yet to reach public sector managers, as evidenced in a recent CIPD study [23] where CEOs of private organizations attached greater importance to talent management than did other sectors such as the public.

Moreover, this same study reflected the problems faced by public sector organizations in retaining professionals and specialists compared to the private sector (60% was the figure indicated by respondents from the public sector compared to 40% from the private sector). This is striking because it also evidences the pressing need to retain talented staff in the public sector. One key element of this study is therefore precisely to bridge such a gap by carrying out a study of talent management and its impact on organizational behavior and service performance (SP) in local government.

Many studies conducted in the field of HR and talent management have attached enormous value to affective commitment towards the organization [24,25]. This commitment has been considered vital in the so-called “soft” models where the focus was placed primarily on the professional development of employees [26]. Recent studies observe how affective organizational commitment (AOC) positively affects job satisfaction and job performance and mediated the effect of work–family conflicts on job satisfaction [27]. Affective commitment acquires particular relevance when linked to dependent variables of the organization, as is the case of service performance in local government. In this way, we relate to both perspectives, “soft” (affective commitment) and “hard” (service performance), as happens in the strategy of most companies [28]. On the other hand, as mentioned before [5], TM is highly contextual, both organizational internal and external context affect the intended TM strategy, including the actors involved in TM and their interrelated logs. These authors suggest a need for deeper, contextually based research contextually, such as on the public sector.

The main goal of this article is to relate TM to the improvement of citizen service through employee commitment. That is to say, we propose that talent management strategies will have a significant impact on service excellence insofar as public employees are committed to the organization.

The present study thus not only advances scientific literature vis-à-vis the need to gain a deep understanding of TM in public administration [5,20] but also proposes a model that relates TM with affective commitment and its final impact on better service provision to citizens. Our research also contributes to the call made by Bertucci [29] towards an improvement in the status and a greater influence of HR managers in public sector organizational structures and decision-making processes.

## 2. Materials and Methods

### 2.1. Materials

#### 2.1.1. Talent Management in Public Administration

The current context of organizations is highly competitive, and talent management is needed to face up to these social, technological and organizational challenges. In fact, what was initially called “the war of talent” in a 1997 study by McKinsey and associates and subsequently published as a book [30], has become such real strategic content that it has been consolidated both professionally and scientifically [31].

The approach adopted in this study is based on the theory of resources and capabilities, where employees are a resource with the potential to be scarce, of value to the organization, and inimitable. This supposes a competitive advantage for the sustainability of the organization over time [32,33]. Indeed, this is the most widely used theory in TM scientific literature [34]. In addition, several studies have already been conducted that analyze mediating variables in the relationship between TM and organizational performance, in an effort to better understand the meaning and interaction of the application of TM in the organization [7,35].

In the case of public services, this performance implies the excellence of the services provided to citizens [36,37,38], although there are few empirical studies exploring this relationship [5]. In fact, of the few studies that can be found, some are theoretical, others use qualitative analysis or are case studies [5,11,12,14,16,17,18,38,39,40,41,42]. Both HR and TM are therefore seen as useful tools for promoting service-oriented behaviors in public servants [43].

In addition, as indicated by Walker and Andrews [44], three quarters of the studies into HR quality and local government performance support the hypothesis that staff quality is an important route for the organization’s success.

However, some voices [16] have alerted as to whether or not these “harder” notions used in the private sector are suited to the public service sector. This is because there is clear tension in public administration in the operationalization of talent management if considered from the exclusionary standpoint [45]. In fact, in many public sector organizations there is a long tradition of a principle of equality [46] that would lean more towards including the principles of TM. This is what Harris and Foster [14] call the “sameness/difference debate”, and is a dilemma that management must face when interpreting and applying HR policies and TM [14].

Our interest focuses on relating TM with the service provided by professionals in a public administration, where talent development strategies (soft) would have an impact on the performance provided by professionals (hard). Thus, HR practices affect employee behavior, stimulating proactivity in public service to citizens [38]. In this way, researchers have long predicted a positive relationship between HR practices and customer service [47]. Such is the case of the so-called “customers’ mindset”, that is, the mentality with which employees treat customers and which is strongly conditioned by how they themselves have been treated by the company [48]. In conclusion, Lawler [49] indicates there is consensus concerning improved company performance when using effective TM.

For all of these reasons, we hypothesize there will be a positive and significant relationship between TM and service performance.

**Hypothesis** **1.** *Talent management is positively and significantly related to service performance: TM → SP.*

HR practices also affect other organizational behaviors, such that the more one invests in employees, the more the employee will be linked to the organization, engaging in a kind of social exchange with the organization [38]. In fact, HR practices affect employee affective commitment [50]. One relevant dependent variable is therefore affective organizational commitment, widely highlighted in the scientific literature [37] and that we link to TM practices. Emotional commitment is an emotional link to the organization because the employee wants the organization to be successful and wishes to feel proud of being a part of the project [51].

As previously mentioned, TM in public sector organizations tends to embrace inclusion policies. Previous studies relate this inclusion to commitment in the organization [52,53,54,55,56,57,58,59,60].

As the most current HR strategy, TM can therefore be conjectured to also have a positive and significant relationship with affective commitment.

**Hypothesis** **2.** *Talent management is positively and significantly related to organizational commitment: TM → AOC.*

#### 2.1.2. Affective Organizational Commitment as a Mediator in the Talent Management and Service Performance Relationship

Organizational commitment has been defined as the “psychological relationship between the employee and his organization that makes it less likely that the employee voluntarily abandons it” [61]. These authors, defenders of the multidimensional vision of organizational commitment, establish three types of commitment: affective, continuance and normative. In the case of affective commitment, the bond is established because employees want to stay in the organization voluntarily due to their identification and emotional relationship. However, the continuity commitment establishes a duty to continue in the organization given the costs involved in abandoning it, while in the normative case the employee must remain through a sense of obligation.

Scientific studies have failed to support this three-dimensional nature because a relationship is seen to exist between normative and affective commitment, whilst there is a lack of clarity in continuance commitment [62]. In fact, affective commitment has received most scientific support, both for its reliability and scientific validity [62,63].

Organizational commitment has been widely studied and related to organizational performance and effectiveness [54], although studies on organizational commitment and its influence on the organization are contradictory if we consider the differences between employees working in public and private organizations [63]. Affective organizational commitment has been related to customer performance, that is, employee performance that meets the expectations of the citizen in public organizations [64,65]. In this way, insofar as employees are more committed, they will display more proactive behavior in order to improve the public services they provide to citizens [37]. Other studies examine how different leadership practices by different leadership roles of public sector managers affect subordinates’ level of affective organizational commitment to the organization. The results show that task-oriented leadership and integrity-oriented leadership strengthen fondness of the organization among those with already high levels of affective organizational commitment, but there is no significance with those members with low level of affective organizational commitment [66].

Despite the different calls to continue research into the public sector, it seems that studies of commitment are still being neglected by the scientific literature [64]. Therefore, we hypothesize that affective organizational commitment will have a significant and positive relationship with the service that employees provide to citizens.

**Hypothesis** **3.** *Affective organizational commitment is positively associated with service performance: AOC → SP.*

As we have seen, affective commitment plays a key role in the organization [26]. This commitment has been considered vital in so-called “soft” models, where the focus is mainly on employees’ professional development [27,28,67]. In fact, affective commitment plays a mediating role in the relationship between HR practices and employee behavior [26].

**Figure 1:**
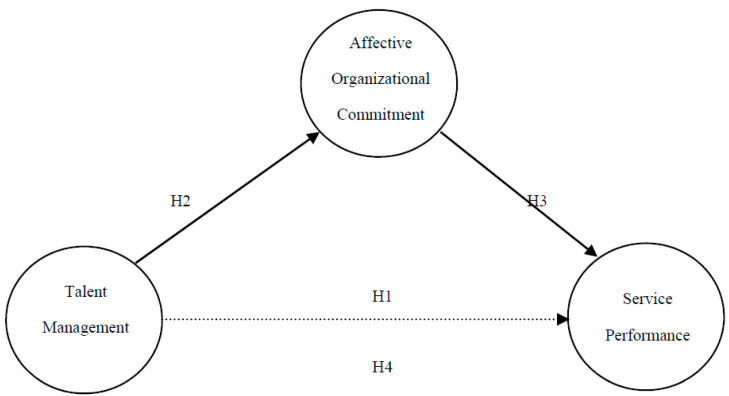
Model proposal: TM → SP mediated by AOC.

For all of the above, we can hypothesize a full model where the relationship between TM and service performance is mediated by affective organizational commitment. This mediation means that by incorporating the variable of affective commitment, it will capture all the variance, leaving no significant relationship between TM and service performance. The model is described in Figure 1.

**Hypothesis** **4.** *Mediator hypothesis: TM → AOC → SP.*

### 2.2. Methods

#### 2.2.1. Sample

Local governments are of great importance to citizens because they represent the closest services. “Local governments are responsible for the management and delivery of key public services in countries worldwide” [44].

The sample used in this study was obtained in 2018 from all the employees of a local government in Spain, which serves a town with a population of almost 10,000 inhabitants. In total, 104 employees answered the questionnaire. In order to guarantee the anonymity of the respondents, no demographic variables were requested. This favored the sincere response of all workers. For this reason, the only descriptive variable was that of their employment relationship with the local administration. Of the total number of respondents, 29.6% were career or interim employees, 27.2% were permanent or temporary employees, and 43.2% were temporary employees.

#### 2.2.2. Measures

Three measures were mainly used to verify the proposed hypotheses: talent management (TM), organizational affective commitment (AOC) and service performance (SP). All three measures used Likert-type scales from 1 to 6: (1) strongly disagree; (2) disagree; (3) slightly disagree; (4) slightly agree; (5) agree; (6) strongly agree.

Table 1 shows the descriptive statistics (mean and standard deviation), composite reliability, extracted variance and correlations between variables. As can be seen, all the measurements show good reliability results.

##### Talent Management

To measure TM, we used four items from previous studies found in the scientific literature [7,35]. The original 5-item questionnaire consisted of: (1) degree of alignment with the organization’s objectives; (2) the development of talent competency that expresses individual feelings of personal mastery and self-efficacy; (3) talent management shown by the managers (this item was excluded when the adjustment models of the measures were analyzed); (4) job autonomy/empowerment; (5) talent management development that expresses the most basic and powerful sense of professional development within the organization.

##### Affective Organizational Commitment

Affective organizational commitment was used as it has received most support in the scientific literature, both for its reliability and validity [63].

To measure affective organizational commitment, an adapted version was used, which consisted of three of the six original items of the revised scale of affective commitment by Meyer and Allen [68]. Specifically, the three items were: (1) I feel a strong sense of belonging to my organization; (2) I feel emotionally attached to the organization; and (3) this organization has great personal meaning for me.

##### Service Performance

Service performance aims to measure the degree to which public employees’ performance is based on the excellence of their services, which in some studies has been called the service mentality [47].

To measure service performance, an adapted version of three items of the Hodgkinson and Hughes [69] measure of customer performance was used. These three items were: (1) This organization gives true customer service; (2) In this organization, we are clear that the priority is the citizen; (3) In this organization, we seek excellence in services.

#### 2.2.3. Analysis

In order to check the mediation between the different variables, we followed the steps specified by Baron and Kenny [70] of the mediating models and Hayes perspective [71]. According Baron and Kenny [70], there must first be a significant relationship between the independent variable and the mediating variable (Hypothesis 2). There must then be a significant relationship between the mediating variable and the dependent variable (Hypothesis 3). Depending on how much these first two significant relationships occur, the significant relationship that existed between the independent variable and the dependent variable (Hypothesis 1) must become non-significant when introducing the mediating variable (Hypothesis 4), indicating a full mediation model.

We also used the Hayes PROCESS macro to determine the significance of the proposed mediation, as well as the Sobel test and the confidence intervals. Indeed, mediation analysis “is used to quantify and examine the direct and indirect pathways through which an antecedent variable X transmits its effects on a consequent variable Y through one or more intermediary or mediator variables” [71]. In line with this author, the PROCESS macro for SPSS estimates direct, indirect effects, errors standard and confidence intervals based on the distribution obtained with the bootstrap method.

## 3. Results

### 3.1. Measurement Models Confirmatory Factor Analysis (CFAs) and Common Method Variance Bias

The different measurement models were specified by CFA. In order to evaluate the measures used, the two-stage analysis process was employed [72]. In this procedure, the measurement model is first proposed using CFA, before the structural analysis model is then developed. Using this method, we analyze the degree to which the different measures obtain a good adjustment model as a single factor model.

In the case of reflective measurement models, the use of composite reliability is suggested (see Table 1) based on the factorial loads of the items in the latent variable [73]. The interpretation is similar to that of Cronbach’s alpha since the value of 0.70 is taken as a minimum in basic research contexts [74]. Of the three measures, two are close to 0.90 showing high values (TM = 0.89 and SP = 0.87) and only one has a value of 0.74 that exceeds the established minimum.

In the same way, in order to guarantee convergent validity, the results of the average variance extracted (AVE) were obtained, where the values must be greater than 0.5. In our case, two variables (TM and SP) are above 0.5. However, AOC is very close to 0.5 (0.48). Fornell and Larcker [75] indicate that values below 0.5 but with a composite reliability above 0.6 also reflect adequate convergent validity.

In order to guarantee discriminant validity, we used the Fornell and Larcker method [75] which establishes that the square root of the AVE must be greater than its correlations with any of the other constructs analyzed. In our case, this condition is also fulfilled for the three variables studied.

As regards the three proposed models, the goodness of fit indexes exceeds the previously established criteria, indicating that they can be used as measures to relate to each other (see Table 2).

Moreover, in order to check the possible common method variance bias in cross-sectional research designs, we have used two main methods: Harman one-factor test [76] and Lindel and Whitney’s method [77]. By testing Harman one-factor test [76] the incidence of variance of the common method can be determined. In fact, through this method an exploratory factor analysis is carried out with all the variables involved analyzing the matrix of factors not rotated. In the event that a single factor emerges or one explains most, it is concluded that the variables are contaminated. In our case, three main factors have been obtained, implying that there are three differential factors and eliminating the possibility of a common variance error. In addition, we also use the method of Lindell and Whitney [77], which includes another variable that is not related but is susceptible to measurement bias. This new variable is initially correlated with all and then with the method of partial correlations to observe if there are changes in the significant differences. In our case, the correlations were maintained with the same significance, indicating the non-existence of the common variance error.

### 3.2. Mediation Testing

As previously mentioned, in order to be able to verify the mediation model, we must first fulfil a series of significant relationships between the variables used. The first of these is the relationship between the independent variable TM and the mediating variable AOC.

Table 3, which analyzes the relationship model between TM and AOC, shows how the goodness of fit indexes exceed the previously established criteria as indicators of a good model. More specifically, the root mean square error of approximation (RMSEA) value is below 0.08, the root mean square residual (RMR) value is below 0.10, and the goodness of fit index (GFI), normed fit index (NFI) and comparative fit index (CFI) values are equal to or greater than 0.950. Similarly, the regression value of the standardized beta which calculates the relationship between TM and AOC is 0.751 (*p* < 0.001), indicating a significant and positive relationship between the two variables. In addition, the variance explained by the coefficient of determination (R^2^) was 56.4%, indicating an adjustment of the model between moderate and substantial.

Table 3 shows the adjustment values of the model that relates the mediator variable to the dependent variable (AOC → SP) where it can be seen how the goodness of fit indexes surpass the previously established criteria as indicators of a good model. More specifically, the RMSEA value is below 0.08, and the GFI, NFI and CFI values are equal to or greater than 0.950. Similarly, the standardized beta value of the estimate of the relationship between AOC and SP is 0.767 (*p* < 0.001), indicating a significant and positive relationship between the two variables. In addition, the explained variance of the model was 64%, indicating moderate to substantial adjustment.

As regards the relationship model between the independent variable (TM) and the dependent variable (SP), it can be seen (Table 3) how the model again presents good adjustment values, and how the standardized beta value of the relationship between TM and SP is 0.535, proving to be significant (*p* < 0.001) and positive.

We can thus proceed to calculate the last model, which is the full model since all the conditions have been fulfilled to be able to establish a mediation model.

The relationship between TM and SP is established in the full model, although the relationship of TM with AOC and AOC with SP is also included. The mediation hypothesis establishes that by incorporating the AOC variable into the model, the prior significant relationship of TM with SP will become non-significant if it is a full mediation model.

**Figure 2:**
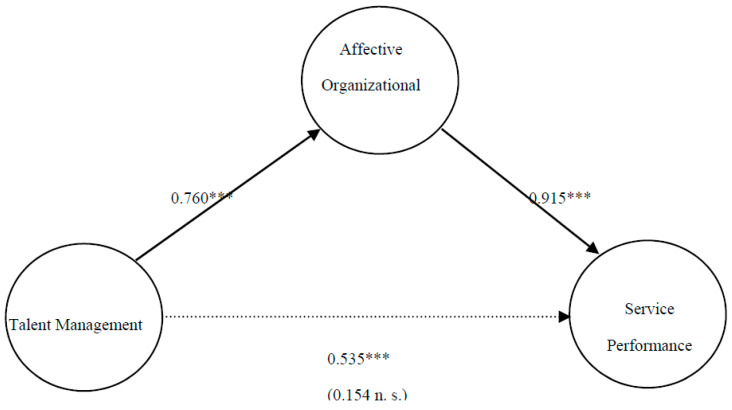
Path analysis: TM → SP mediated by AOC. Note: *** *p* < 0.001.

In our case, the results shown in Table 3 and Figure 2 indicate that the proposed model meets the minimum standards for a good model. In addition, the previous relationship between TM → SP, which was significant (*p* < 0.001), has become non-significant, thus confirming a full mediation model.

The model (Figure 2) shows how the relationship between TM → AOC has a standardized beta value of 0.760 (*p* < 0.001) and the relationship between AOC → SP has a standardized beta value of 0.915 (*p* < 0.001). As regards the coefficient of determination, the results show that the explained variance of the SP dependent variable was 64.7%, being between the adjustment levels of the model considered to be moderate and substantial. In addition, the AOC variable obtained an explained variance of 57.7%, being at the same level as the previous variable. In both cases, the model thus has sufficient predictive capacity according to the criteria of Falk and Miller [78], with its value indicating the model’s degree of accuracy [73].

The Sobel test was used to analyze the significance of the mediator model, with a significant z-value indicating there is a significant reduction caused by the mediating model. The results of the Sobel test support the hypothesis of the assumption of a two-tailed z-test that the effect of mediation equals zero. In the Sobel version (Z = 4.06, *p* < 0.001), the Aroian version (Z = 4.37, *p* < 0.001), and the Goodman version (Z = 4.43; *p* < 0.0001), the results indicate a significant reduction in the mediating model, corroborating our hypothesis.

Given the criticisms received by Baron and Kenny’s approach regarding the indirect effect of mediation [71,79], and to give more solidity to the results, the Hayes PROCESS method was also used with the proposed macro using the SPSS. First, the results of the multiple linear regression analysis are presented, where the positive and significant impact of the independent talent management variable on the organizational commitment mediating variable (standardized β = 0.59; t = 7.51; *p* < 0.001) is analyzed, with a percentage of variance of 35.61%. Second, the model is analyzed where the two TM and AOC variables are the predictor variables of the service performance variable in a multiple linear regression model. The results show a significant and positive value both in TM (standardized β = 0.24; t = 2.51; *p* < 0.01) and in AOC (standardized β = 0.46; t = 4.83; *p* < 0.001). This model explains 40.44%.

Third, the total effect of the TM variable on the SP is presented (standardized β = 0.51; t = 6.08; *p* < 0.001) being significant and positive. Fourth, we show the direct effect of the independent variable TM on the dependent variable SP (standardized β = 0.25; t = 2.51; *p* < 0.01) being significant and positive. Finally, we show the indirect effect of TM on SP mediated by the mediating variable AOC (β = 0.28; t = 2.51; *p* < 0.01), where the predictive value is significant and positive. Furthermore, to verify the significance of partial mediation with the bootstrap method, the confidence interval values should not contain the zero value (0.1464–0.4232), as is the case in our results. We can therefore confirm the hypothesis of the mediation of our model under the model of Baron and Kenny, as well as under the perspective of the Hayes method.

In order to avoid the problem of endogeneity, a control variable was used: satisfaction with wages. The results of the new model indicate that this variable did not show significant values (β = 0.01; t = 0.11; *p* < 0.91) on the dependent variable. Furthermore, the general model kept the same values of beta and significance as the previous one without the control variable.

## 4. Discussion

For public administration, finding the right people who will provide organizational success is critical. To achieve this, internal reforms involving improvement of services are required [11]. TM is thus key in new public management (NPM), where the role of employees’ affective commitment must be considered vital. In fact, as long as affective commitment is generated, employees will seek excellence in the service they provide to citizens. Thus, two main goals are achieved: first a more active role in the quality of service provided by employees and, secondly, affective employee involvement with the organization. Both aspects are critical in modernizing public administration and its expected results of improvement and success in organizations.

### 4.1. Theoretical Implications

The present study proves valuable because it analyzes a first order variable in public administration, service performance, and more importantly, establishes a mediating model which explains how this improvement in professionalism can be provided.

From a theoretical point of view, and in line with the theory of resources and capabilities [32,33], it is precisely human capital that makes the difference in competitiveness and organizational success. In our case, the proposed mediator model informs us that in order to achieve service excellence, we need employees’ emotional commitment. Achieving employees’ affective commitment seems to be key to them providing us with service excellence, a commitment which is achieved by applying TM strategies. Therefore, as long as employees develop their talent in the organization (soft HR), they will achieve an emotional commitment which will drive their service performance excellence (hard HR) [25,28,38].

The full-mediating model obtained in this article again raises the classical debate in HR literature concerning soft and hard strategies [26,28,38,66] by placing the emphasis on an integrative model that links a clear “soft” orientation with organizational results. As already mentioned in Trust et al. [28], real and applied models mix the two perspectives.

Likewise, the debate in the scientific literature concerning inclusion, as previously mentioned, is vital vis-à-vis addressing the application of TM in public organizations. This aspect has been shown in the present article, since it has already been demonstrated that inclusion strategies, where all employees display their talent, generate organizational results at all levels.

### 4.2. Managerial Implications

Over the last few years, public administration has been undergoing constant transformation, not only due to changes in the environment, but also because of the need to adopt the principles of NPM. This involves a concern for improving services through the professionals who provide them, which in turn entails managing people in a way that is different, more flexible, less bureaucratic, and more client- or citizen-oriented. All of these changes are affecting the management strategies and practices which seek to achieve efficiency and organizational improvement, although what impact this is actually having on local government performance is not yet clear [44].

Our study confirms that applying development strategies for all employees implies better results in service performance. In fact, if an inclusion strategy is generated in talent management, the impact on employee commitment is clear [54]. Alternatively, it may be possible to establish an integrated model of inclusion and exclusion in public administration [15].

What does seem clear is that public employees must develop a “customer mindset” [48], while at the same time TM is demanding a “talent mindset” [7,30]. From both perspectives, there needs to be an extension of the mentality that either focuses on development and learning (soft) or on results (hard). Not only will it have an impact on greater employee commitment, but it will ultimately benefit the citizen, who is the one mainly receiving the services of local government.

Furthermore, any reforms and transformations imply an evolution in the meaning of professionalism and in the identity forged by those working in public administration [80]. As pointed out, changes must be made both in the structures and strategies, as well as in the mindset of the professionals involved. This is the only way to deal with citizens’ shifting expectations with regard to execution times, service performance and the quality of the services they receive, all of which has had to merge with the budget cuts implemented in recent years [81].

### 4.3. Future Research

Much research remains to be done in the public service environment, since there seems to be more theory than reality, and even more so when talking about management, HR and TM. Although studies tend to transfer management models directly from private companies, it is clear that greater integration with the public function is needed and that direct transfer and application do not prove beneficial. Many more studies are thus needed that can better contextualize private sector progress in the public management sphere, both in TM and in the study of affective commitment and service performance.

Nevertheless, progress has been made in answering some questions which we hope may be supported by other researchers. Such is the case of how the inclusion strategy is integrated with TM. Yet, as suggested in the literature, can an integrative model be considered? Or does this, on the other hand, entail implementing only inclusive talent models in the public sector? More empirical studies are needed in mixed models where the impact of exclusion in inclusion models is tested; in other words, where working more intensely with top management is combined with developing talent, albeit perhaps less intensely in the rest of the organization. In the same way, we need to better understand how TM strategies can be transformed and adapted to the current reality of the public function.

In addition, more studies are needed with mediating variables that allow us to better understand organizational results in their connection with TM in public organizations. This necessary integration between the “soft” and the “hard” in HR is also a challenge for research in public sector TM.

### 4.4. Research Limitations

Certain limitations in this study are common to much of the current literature in that a sample in a specific population is used. Studies in other locations and in other types of public administration are required if the findings to emerge here regarding the benefits of TM and the importance of staff quality in organizational success are to be generalized [44].

In addition, the measures must be replicated in other studies in order to substantiate the reliability and validity statistics. In fact, the measures used here are subjective and must be complemented by other types of objective measures so as to better understand the phenomena studied. In addition, the measures are self-report questionnaires which may generate common-method bias [82,83].

Moreover, this is a cross-sectional design study. Longitudinal designs should also be considered, especially in studies with causal relationships. The use of measures at different times is thus suggested, particularly when talking about independent and dependent variables where certain variables have a causal relationship with the others.

## 5. Conclusions

This research examines the relationship that occurs when high-performance talent management practices are implemented over service performance in a public service. In fact, a main conclusion is that the affective organizational commitment is essential to understand this relationship because it is a total mediator in the relationship. This allows us to better understand the relationship between talent management and performance variables incorporating the variable affective organizational commitment.

Another conclusion, and related to the first, is that public organizations, apart from establishing intervention frameworks based on talent management, have to work intensively on the affective commitment of their employees to achieve greater productivity. This implies strategies focused on attracting, developing and retaining employees capable of generating an effective organizational commitment.

## Figures and Tables

**Table 1:** Descriptive statistics, reliability and correlation coefficients.

Variable	Mean	Standard Deviation	Composite Reliability	Average Variance Extracted	1	2	3
1. Talent Management (TM, 4 items)	4.05	1.27	0.89	0.67	1		
2. Affective Organizational Commitment (AOC, 3 items)	3.42	1.19	0.74	0.48	0.597 **	1	
3. Service Performance (SP, 3 items)	3.65	1.33	0.87	0.69	0.516 **	0.606 **	1

** *p* < 0.01 (two-tailed tests) (scales measured from 1 strongly disagree to 6 strongly agree).

**Table 2:** Adjustment indices of the confirmatory factor analysis of the three variables.

	χ^2^ (d.f.)	RMR	RMSEA	GFI	NFI	CFI
**TM Confirmatory Factor Analysis Model**						
Model 1: independence model	249.053 (6)	1.116	0.627	0.422	0	0
Model 2: model proposal	3.819 (2)	0.051	0.094	0.982	0.985	0.993
**AOC Confirmatory Factor Analysis Model**						
Model 1: independence model	66.600 (3)	0.723	0.454	0.688	0	0
Model 2: model proposal	0 (0)	0.000	0.000	1.000	1.000	1.000
**SP Confirmatory Factor Analysis Model**						
Model 1: independence model	161.448 (3)	1.091	0.716	0.514	0	0
Model 2: model proposal	0 (0)	0.000	0.000	1.000	1.000	1.000

Note. d.f. = degrees of freedom; RMR = root mean square residual; RMSEA = root mean square error of approximation; GFI = goodness of fit index; NFI = normed fit index; CFI = comparative fit index.

**Table 3:** Fit indices of the path analysis.

	χ^2^ (d.f.)	RMR	RMSEA	GFI	NFI	CFI
**TM → AOC Model**						
Model 1: independence model	380.811 (21)	0.958	0.408	0.382	0	0
Model 2: model proposal	19.257 (13)	0.080	0.068	0.950	0.950	0.983
**AOC → SP Model**						
Model 1: independence model	297.767 (15)	0.948	0.428	0.432	0.000	0.000
Model 2: model proposal	6.700 (7)	0.063	0.000	0.980	0.977	1.000
**TM → SP Model**						
Model 1: independence model	458.572 (21)	1.014	0.450	0.363	0	0
Model 2: model proposal	17.301 (12)	0.094	0.065	0.955	0.962	0.988
**TM → SP mediated by AOC Model**						
Model 1: independence model	637.637 (45)	0.959	0.358	0.314	0	0
Model 2: model proposal	41.074 (30)	0.095	0.060	0.932	0.936	0.981

Note. d.f. = degrees of freedom; RMR = root mean square residual; RMSEA = root mean square error of approximation; GFI = goodness of fit index; NFI = normed fit index; CFI = comparative fit index.
